# The Impact of Diet Protein and Carbohydrate on Select Life-History Traits of The Black Soldier Fly *Hermetia illucens* (L.) (Diptera: Stratiomyidae)

**DOI:** 10.3390/insects8020056

**Published:** 2017-05-31

**Authors:** Jonathan A. Cammack, Jeffery K. Tomberlin

**Affiliations:** Department of Entomology, Texas A&M University, College Station, TX 77843, USA; jktomberlin@tamu.edu

**Keywords:** artificial diet, mass rearing, alternate protein production, nutrition ecology

## Abstract

This study examined the impact of diet protein and carbohydrate percentages as well as moisture on the immature development, survivorship, and resulting adult longevity and egg production of the black soldier fly, *Hermetia* *illucens* (L.) (Diptera: Stratiomyidae). Moisture impacted development and corresponding life-history traits more than protein:carbohydrate content; larvae were unable to develop on diets at 40% moisture. Larvae fed diets at 70% moisture developed faster, grew larger, and required less food than those reared on diets at 55% moisture. Larvae reared on the balanced diet (21% protein:21% carbohydrate) at 70% moisture developed the fastest on the least amount of food and had the greatest survivorship to the prepupal stage. Adult emergence and longevity were similar across treatments, indicating immature life-history traits were impacted the most. The control (Gainesville house fly) diet was superior to the artificial diets for all parameters tested. These differences could indicate that other constituents (e.g., associated microbes) serve a role in black soldier fly development. These data are valuable for industrialization of this insect as a “green” technology for recycling organic waste, which can be highly variable, to produce protein for use as feed in the livestock, poultry, and aquaculture industries, as well as for bioenergy production.

## 1. Introduction

As the human population increases, so too does the amount of waste generated. In the year 2000, approximately 49% of the world population lived in cities and generated more than three million metric tons of waste (e.g., household items, food waste, packaging, ash) on a daily basis [1]; by 2025, this number is expected to double. The FAO estimates 1.6 Gtonnes of food waste were generated worldwide in 2007, which accounted for approximately one third of global food production, and this waste occurs at all stages from production to consumption [2]. In addition to taking up space in landfills, contributing to the spread of pathogens, and the production of noxious odors, food waste is also the number three contributor of global CO_2_ production and produces more than double the CO_2_ produced by all ground transportation in the United States [2]. For several decades, researchers worldwide have proposed using the black soldier fly, *Hermetia illucens* (L.) (Diptera: Stratiomyidae), as a means to process organic matter such as food waste to divert these materials away from landfills.

The black soldier fly is a wasp-like fly distributed throughout the tropic and temperate regions of the world. The species is native to the new world, but through human-mediated dispersal, is now found almost globally [3,4]. What is truly unique about this species is its ability to successfully colonize a wide variety of resources ranging from bananas [5], swine remains [6], human remains [7,8] and fish offal [9], to food waste [10,11,12], as well as human [13] and livestock feces [14,15,16]. Additionally, research on bioconversion of poultry manure indicates that diets with moisture contents between 40% and 70% are optimal for soldier fly development [16]. Because of its general nature as a consumer of various decomposing materials and ability to be mass-produced [17], this species has been studied as a sustainable means for recycling nutrients in organic waste [18,19]. Previous research efforts have demonstrated that the larvae of this species can convert 50% of waste dry matter into insect biomass high in protein and fat [19], which can be used to develop feed for the aquaculture [20,21,22], livestock [23], and poultry [24,25] industries (see Makkar et al. [26] for a review). Recently, the Association of American Feed Control Officials approved the use of whole black soldier fly larvae as a feed for farm-raised salmonid fish, representing the first approval of using an insect for animal feed in the United States [27]. Furthermore, the fat from the larvae can be extracted and used for biodiesel production [28,29], and the chitin and its derivatives have various uses in medical and pharmaceutical applications [30], as well as a number of industrial usages including food processing and packaging, cosmetics, textiles, and agriculture [31].

A challenge facing the optimization and implementation of this system lies in the heterogeneous nature, such as nutrient and moisture content, of organic waste material. For example, restaurant waste (containing animal and plant matter) is approximately 20% protein, 20% fat, and 57% carbohydrate, whereas mixed fruits and vegetables are approximately 20% protein, 2% fat, and 69% carbohydrate (see Table 1, modified from Nguyen et al. [11]). Heterogeneous resources are not unique to decomposing organic matter and waste management; many herbivorous insects face this same challenge in nature. Much research has been conducted on the ability of these insects to acquire optimal nutrition and the subsequent effects on life-history and fitness, as plants are nutritionally heterogeneous in both space and time [32]. As reviewed by Deans et al. [32], caterpillars (Lepidoptera) and grasshopper nymphs (Orthoptera) consuming higher proportions of protein to carbohydrates develop faster, but are leaner and have a lower mass. Variation in micronutrients has been shown to impact community abundance and assemblage of herbivorous insects within given habitats [33]. Furthermore, previous work has well established that many insects can be selective with regards to what, and how much, they consume [32,34,35]. 

As previously mentioned, a current hurdle to the application of black soldier flies to organic waste management is that the factors limiting or regulating soldier fly development on different waste streams are not known. Once these factors are determined, waste streams could be manipulated as a means to optimize waste reduction and conversion of these materials to insect biomass (i.e., protein and fat). Therefore, the objectives of this study were to determine the impact of protein:carbohydrate percentages and diet moisture on life-history traits of the black soldier fly.

## 2. Materials and Methods

### 2.1. Insect Colony

The black soldier fly colony used in these experiments was established in January 2014 from eggs received from Phoenix Worm, Inc., Tifton, GA, USA, which was initiated from a laboratory colony at the Coastal Plains Experiment Station, University of Georgia, also located in Tifton, GA, USA. This colony has been maintained for 18 years and supplemented periodically with wild-caught material.

Flies were reared following a modified version of the methods outlined in Sheppard et al. [17]. The adult colony was housed in a mesh tent (1.4 (L) × 1.4 (W) × 1.8 (H) m; 1.5 mm mesh) in a greenhouse at approximately 27−30 °C and 70% relative humidity (RH). Eggs were collected twice weekly in 5 (L) × 3 (W) × 3 (H) cm triple-layer corrugated cardboard egg traps. To stimulate oviposition, the colony was presented with a 5.67-L Sterilite^®^ shoe box containing approximately 500 g of saturated Gainesville diet (50% wheat bran, 30% alfalfa meal, 20% corn meal [36]) and 500 black soldier fly larvae. To prevent flies from contacting the media, the shoe box lid had a 7 × 12 cm screened opening on which the cardboard egg traps were placed. Once eggs were collected, a 1.0-g aliquot was placed in a 30-mL cup with a paper lid (Bio-Serv™, Flemington, NJ, USA), which was placed inside a Choice 480 mL deli cup (WebstaurantStore Food Service and Equipment Supply Company, Lancaster, PA, USA) covered with a paper towel and rubber band. This container was held in a Rheem Environmental Chamber at 27 °C, 55% RH, on a 14L:10D cycle, and monitored daily for eclosion. Resulting larvae were maintained under the same environmental conditions on the Gainesville diet, hydrated to 70% moisture by weight.

For experiments, eggs were collected in the same manner, but the cardboard egg traps were presented to the colony for a maximum of 24 h. Eggs were monitored daily for eclosion, after which larvae were provided with 10 g of moistened Gainesville diet, prepared as previously described. From the age of 2–7 days, larvae were provided with 10 g of diet daily. Experiments were started with 8-day-old larvae.

### 2.2. Artificial Diet Experiments

Dry, chemically-defined, cellulose-based diets were prepared following methods similar to Simpson and Abisgold [35] and Behmer et al. [37]. Protein and carbohydrate components in the diets were varied to give protein:carbohydrate percentages (presented in percentage dry weight) of 7:35, 21:21 and 35:7, resulting in total macronutrient content of 42%. After preparation, the powdered diets were stored frozen at −20 °C. When needed for feeding to the larvae, batches of diet were mixed with water and distributed among each container receiving that diet-moisture treatment to ensure each replicate container received the same diet. Diets were hydrated to 40, 55 or 70% moisture by weight, resulting in nine experiment treatments; Gainesville diet hydrated to 70% moisture served as a control. Four replicates of each diet-moisture were used in each trial (40 total containers); two trials of the study were conducted.

For experiments, larvae were reared in 720-mL Reditainer™ deli containers covered with a 15 × 15 cm WypAll* wiper (Kimberly-Clark Global Sales, LLC, Roswell, GA, USA) and rubber band to prevent contamination, in a Percival I-36LLVL incubator set at 30 °C, 70% RH, on a 14L:10D cycle. At 8-days-old, approximately 125 larvae were placed in the appropriately-labeled 720-mL rearing container with 10 g of the respective diet treatment. Larvae receiving the control diet were fed daily, and larvae receiving test diets were fed every two days for the duration of the experiment and were monitored daily.

Prepupae were removed daily from each container and weighed, then placed in appropriately-labeled 720-mL rearing containers, containing approximately 13.5 g of vermiculite, which provided a 2.5 cm-deep pupation substrate. Once 40% of the larvae within a given container reached the prepupal stage, the addition of diet ceased [38]. Once five consecutive days had passed without the collection of a prepupa from a given container, monitoring of that container ceased, and remaining larvae were counted and classified as moribund. Prepupae/pupae were held in the same incubator in which the larvae were reared and were monitored daily for adult emergence. Upon adult emergence, the first two male and female flies to emerge from each replicate container were placed in individual Dart^®^ 37.5-mL plastic soufflé cups (Dart Container Corporation, Mason, MI, USA) with lids, and the flies were monitored daily for longevity. A cotton wick was inserted through the lid so that the flies could be provided with water, three times daily. The remaining emerged adults were released into 84 (L) × 84 (W) × 133 (H) cm cages (Insect-A-Hide™ pop-up shelter, Lee Valley Tools, Ltd., Ogdensburg, NY, USA) in the greenhouse previously described (one cage per diet-moisture treatment) and monitored for egg production, using methods as described above.

When handling larvae or prepupae, and hydrating and aliquoting diets, all items (e.g., forceps, diet mixing cup, and spatula) were wiped with 10% bleach and 70% ethanol to sterilize. All activities regarding observation, larval handling and feeding were conducted using the sterile technique in a biosafety cabinet to prevent contamination of the individual rearing containers.

### 2.3. Data Analysis

A nested analysis was used to determine the impact of diet protein:carbohydrate and moisture content on life-history traits of the black soldier fly. Amount of diet provided, time (days) required for 40% prepupation, prepupal size, adult longevity, and egg production were compared across all diet-moisture treatments using analysis of variance followed by the Tukey–Kramer HSD test, and trial differences (significance set at *p* < 0.05) were tested using the paired *t*-test. Interactions between diet protein:carbohydrate, diet moisture, and trial on each life-history parameter were also examined. All analyses were done in JMP^®^ Pro, Version 12.0.0 (SAS Institute Inc. 2015, Cary, NC, USA). 

## 3. Results

**Figure 1:**
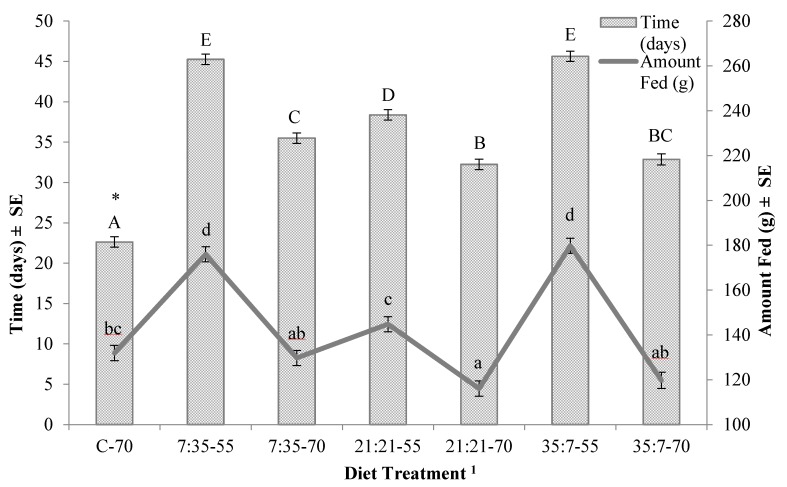
Feeding duration (days) and amount of feed (g) required for 40% of the black soldier fly larvae feeding on a diet treatment to reach the prepupal stage in an incubator set at 30°C, 70% RH, on a 14L:10D cycle. Uppercase letters indicate significant differences in duration of the feeding stage, and lowercase letters indicate significant differences in the amount of feed required for 40% of the larvae to reach the prepupal stage (ANOVA followed by Tukey–Kramer HSD, α = 0.05). ^1^ Treatments are presented as protein:carbohydrate-moisture; * indicates significant difference in duration between trials.

**Figure 2:**
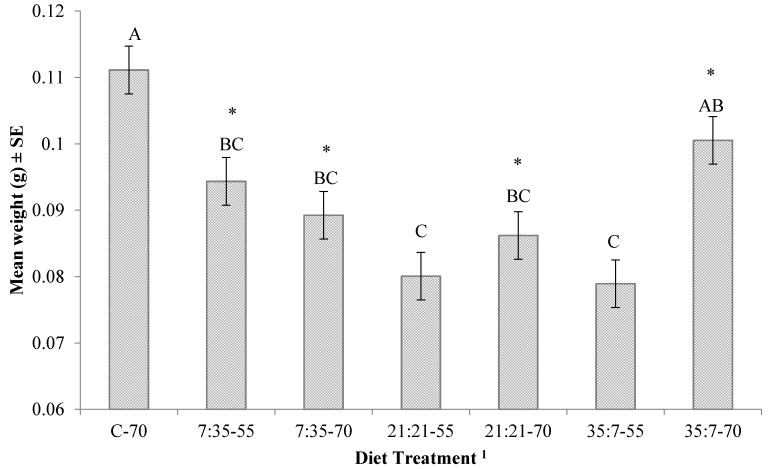
Mean weight (g) of black soldier fly prepupae produced on a diet treatment in an incubator set at 30 °C, 70% RH, on a 14L:10D cycle. Letters indicate significant differences (ANOVA followed by Tukey–Kramer HSD, α = 0.05). ^1^ Treatments are presented as protein:carbohydrate-moisture; * indicates significant difference between trials.

There was not a significant trial by diet treatment (protein:carbohydrate-moisture) interaction for any life-history parameter associated with immature black soldier flies. There was a significant interaction between protein:carbohydrate and moisture content for amount of diet required for 40% of the larvae to reach the prepupal stage (*p* = 0.0002) (Figure 1), time to 40% pupation (*p* < 0.0001) (Figure 1) and mean prepupal weight (*p* = 0.0055) (Figure 2). Diet significantly impacted (F_6,54_ = 154.732, *p* < 0.001) the duration of the feeding stage and, in general, was negatively related to moisture. Larvae reared on diets at 70% moisture developed more quickly (by 6–10 days) and required 25–50% less diet than those fed at 55% moisture (Figure 1); larvae failed to develop on diets at 40% moisture. Trial had no effect on duration of the feeding stage for the test diets, but did for larvae fed the control diet; those reared on the control diet reached the prepupal stage significantly more quickly in Trial 2 (1.75 day, or ~8% faster; *t*(6) = −3.13, *p* = 0.020). Additionally, in the second trial, prepupae were significantly heavier by 12–22% (0.012–0.019 g) (*t*(54) = 2.698, *p* = 0.009) when reared on four of the seven diets that yielded prepupae.

**Figure 3:**
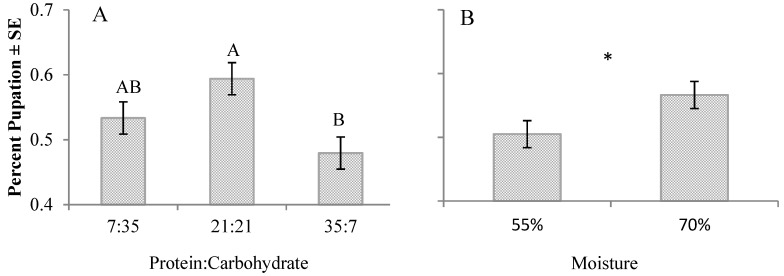
(**A**) Effects of diet protein:carbohydrate on percent pupation. (**B**) Effects of diet moisture on percent pupation. Letters (ANOVA followed by Tukey–Kramer HSD) or asterisk (*t*-test) indicate significant differences (α = 0.05) for each corresponding diet parameter.

**Figure 4:**
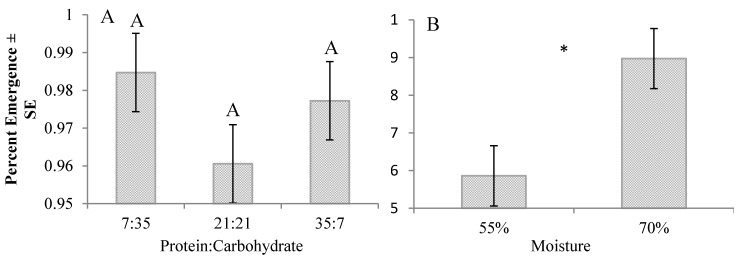
(**A**) Effects of diet protein:carbohydrate on percent adult emergence. (**B**) Effects of diet moisture on percent adult emergence. Letters (ANOVA followed by Tukey–Kramer HSD) or asterisk (*t*-test) indicate significant differences (α = 0.05) for each corresponding diet parameter.

There was no significant interaction between protein:carbohydrate and moisture content for percent pupation (Figure 3A,B) or percent adult emergence (Figure 4A,B). Percent pupation was significantly higher on the 21:21 diet (higher by 11–33%) (F_2,45_ = 5.37, *p* = 0.0081) and on 70% moisture diets (higher by 14%) (*t*(46) = 2.04, *p* = 0.0471). Percent pupation significantly differed between trials only for those reared on the 21:21 diet at 70% moisture (*t*(6) = −4.410, *p* = 0.004); significantly more prepupae (18%) were produced on this diet in the first trial. Protein:carbohydrate content had no effect on adult emergence (*p* = 0.2516), but significantly more (3%) adults emerged when reared on 70% moisture diets (*t*(46) = 2.75, *p* = 0.0084).

Adult longevity data are not presented due to inconsistent and inconclusive results (there were significant interactions between diet treatment and trial, *p* = 0.013). However, longevity ranged from 4.6–6.9 days. Additionally, due to inconsistent oviposition across treatments and trials, these data are also not presented; egg collections ranged from 0.32–3.08 g.

## 4. Discussion

This study is the first to investigate how varying the content of two macronutrients, protein and carbohydrate, in an artificial diet at different moisture levels impacts the life-history of the black soldier fly. Additionally, we summarize some past studies and offer a comparison to the results generated in this study (Table 2, Table 3 and Table 4). These data provide valuable insights into the generalist nature of the black soldier fly and its ability to utilize a variety of resources for larval development. Larvae feeding on carbohydrate-biased diets developed slower (by 0.375–6.875 days) than those feeding on the other diets, which supports findings from previous studies conducted on noctuid caterpillars (Lepidoptera: Noctuidae) [32,39] and acridid grasshopper nymphs (Orthoptera: Acrididae) [34] fed diets similar to those used in the current study. Nash and Chapman [40] also found that larvae of the Mediterranean fruit fly, *Ceratitis capitata* (Wiedemann) (Diptera: Tephritidae), developed significantly slower when reared on a low-protein diet (content or quality) when carbohydrate content remained constant, suggesting protein was the key nutrient for larval development. Oonincx et al. [12] recorded the fastest development and greatest survival rate when black soldier fly larvae were reared on a food waste diet high in protein (22%) and high in fat (9.5%); however, the carbohydrate content of that diet is unknown. In the current study, the development rate was faster, and larval survival was greater (range: 32.250–38.375 days and 57–62%, respectively) on the protein:carbohydrate balanced diet (21:21). Although we saw a difference in prepupal size between trials, this is likely a result of lower survival in Trial 2, as more diet per larva would have been available.

Development rate, prepupal size, and survival, as well as adult emergence were all influenced by moisture. For the two highest protein diets, each of these life-history traits was positively affected by higher moisture content. However, this is contradictory to the findings of Fatchurochim et al. [16], where adult emergence and development rate of the black soldier fly were lower when fed poultry manure at 70% moisture, in comparison to those reared on poultry manure at 40, 50, or 60% moisture. The diets used by Oonincx et al. [12] were at least 67% moisture when fed to the larvae (diet materials were freeze dried and rehydrated with 2 g water per 1 g diet), and larval survival was 72% or greater for all diets tested.

In this study, percent emergence for adults produced from the Gainesville diet was higher than that reported by Tomberlin et al. [38], but adult longevity was shorter. Adult emergence in our study was 97.35% in comparison to 27.2%, and adults fed Gainesville diet lived 6.94 days in comparison to 7.9–9.3 days for females and males, respectively, in the aforementioned study. Adult longevity was also lower than that reported by Tomberlin et al. [42] by 5.46–8.96 days, when larvae were reared on the Gainesville diet; 1.56–2.76 days shorter than when reared on Chemical Specialties Manufacturers’ Association fly diet (Chemical Specialties Manufacturers’ Association, Ralston Purina, St. Louis, MO, USA) or layer hen ration [38]; and 3.02–5.37 days shorter than when reared on dairy manure [14].

Although no conclusive effects of protein and carbohydrate on adult longevity were observed in our study, previous research indicates that diet protein is important for other insect species. Adult longevity of the banana stalk fly, *Derocephalus angusticollis* Enderlein (Diptera: Neriidae), was longest when larvae were reared on diets containing intermediate levels of protein (11 g/L) in comparison to low- (2.7 g/L) or high-protein (33 g/L) diets [43], supporting previous research that found high-protein diets to be toxic to the species [44]. Li et al. [45] determined adult longevity in the honey bee *Apis mellifera ligustica* Spinola (Hymenoptera: Apidae) was dependent on protein content of the larval diet. Those fed higher-protein diets (25% and 35%) lived significantly longer than those fed low protein (15%) diets. However, adult longevity was not significantly different between the two higher-protein diets, suggesting that a diet that is 25% protein might be optimal for honey bee survival. 

Egg production in our experiments was likely low due to the long duration of adult emergence. In Trial 1, adults eclosed over a range of 13–34 days, and in Trial 2, adults eclosed over a range of 15–35 days, depending on diet treatment. The lower numbers in both ranges (13 and 15 days) represent those fed the control diet, from which the most eggs were collected; and the higher numbers represent the 7:35–55 diet. Likely by the time that sufficient numbers of adults had emerged and were present in the cages for mating (particularly the 55% moisture diets), many of the adults were old or had already died. Such information (i.e., predicting emergence patterns) could prove critical in mass production, as maintenance of an adult colony is required for producing larvae for waste digestion.

Although development was generally slower for larvae fed our diets than that found in previous studies (see Table 2), a potential explanation could be differences in daily feed rate. Larvae in our study received 0.08 g/larva/2 days (test diets), whereas larvae fed dairy manure in the Myers et al. [14] study received 0.09 g/larva/day. Unfortunately, the feed rate used by Nguyen et al. [41] varied throughout the study, and Oonincx et al. [12] fed ad libitum, so we are not able to draw any comparisons based on feed rate. Our larvae feeding on the Gainesville diet received 0.08 g/larva/day (0.01 g/larva/day less than provided by Myers et al. [14]), but in general developed more quickly than larvae in the studies by Myers et al. [14], Nguyen et al [41] and Oonincx et al. [12]; we hypothesize that this difference is due to the rearing temperature: our larvae developed at 30 °C, whereas larvae in the previous studies were reared at 28, 27 and 28 °C, respectively. 

We determined moisture content in combination with diet protein and carbohydrate content influenced larval development and survivorship to the adult stage. Such information could prove valuable with industrialization of this insect as a “green” technology for waste recycling to produce protein as a feed substrate for livestock, poultry and the aquaculture industries, as well as bioenergy production. Previous research on the black soldier fly as a waste-management tool has focused on reducing specific wastes, such as manure [14,15], food waste [11,12] and animal rendering by-products [9]. Although such a method is useful for waste reduction, it is not practical for alternate protein production. The nutrient content of such wastes varies greatly (see Table 1), with protein:carbohydrate ratios ranging from 1:2–91:1, resulting in variable nutrient content of the prepupae produced from these materials.

Mixing waste types to optimize dry matter reduction and growth and development of black soldier fly larvae should be investigated further. To simulate a real-world scenario of waste management for insect protein production, Oonincx et al. [12] fed black soldier fly larvae mixtures of waste/by-products from the food production industry, but observed little variation in nutrient content of the larvae. St-Hilaire et al. [9] found that mixing wastes in different ratios (dairy cow manure and fish offal) resulted in prepupae that were significantly larger than when reared on dairy manure alone and that the fatty acid content of these prepupae differed depending on the ratio of the two waste types. For the aquaculture industry, this is an important consideration, as numerous studies have investigated the use of black soldier fly products as a partial or whole replacement for fish-based products in the diets of farm-raised fish (see Makkar et al. [26] for a review). Different species of fish have different fatty-acid requirements for development (see Tocher [46] for a review); in order to fully utilize the potential of black soldier fly products in aquaculture production, the diets containing these products must be equal to that containing fish meal/oil to prevent negative impacts on growth. However, changes in legislature in countries worldwide must occur so these methods can be employed and reduce pressures on global marine fisheries [47].

Future research should employ choice tests with the geometric framework [48,49] to assess decision-making by black soldier fly larvae and its relationship to optimized development. Larvae might require different nutrient ratios for optimal development as they age. Additionally, this study was conducted on a population of black soldier flies that has been in colony for a long time and might not be representative of the response of black soldier flies in the wild or colonies established from different populations around the world. From an applied standpoint, research should follow the work of St-Hilaire et al. [9], Oonincx et al. [12] and ur Rehman et al. [50] and focus on nutrition ecology of mixing different waste types to optimize waste reduction, soldier fly growth and subsequent protein production.

## 5. Conclusions

Due to their generalist nature and high nutritional content, larvae of the black soldier fly have been proposed as a means to address two global issues: organic waste management, and alternate protein production. In this study, we examined the impact of diet protein and carbohydrate percentages and moisture on the life history of black soldier flies. Moisture had the greatest impact on life history: at 40% moisture larvae failed to develop, and those fed diets at 70% moisture developed faster and grew larger than those fed diets at 55% moisture. Larvae reared on the diet containing 21% protein and 21% carbohydrate, hydrated to 70% moisture, developed the fastest and had the greatest survival. Additionally, diet had little to no impact on adult life-history traits. Future applied research on this species should focus on combining waste streams to create a resource that maximizes waste reduction and optimizes black soldier fly production.

## Figures and Tables

**Table 1:** Nutrient content of five different waste streams and black soldier fly prepupae produced from each resource. Modified from Nguyen et al. [11].

	Quantity/100 g	Poultry Feed	Swine Liver	Swine Manure	Restaurant Waste	Fruits and Vegetables	Fish Rendering
Waste Stream	Calories	310.48	442.68	295.23	484.32	375	502.76
	Protein (g)	18.02	76.71	22.66	20.41	20.07	50
	Fat (g)	2.52	12.84	1.4	19.58	1.55	36.18
	Carbohydrate (g)	53.62	4.74	47.61	56.79	68.95	0.55
	Protein:carbohydrate	1:2.97	16.18:1	1:2.1	1:2.78	1:3.44	90.9:1
Resulting BSF * Prepupae	Calories	130	214	−	−	105	233
	Protein (g)	14.7	21	−	21.2	12.9	19.4
	Fat (g)	4.02	8.39	−	−	2.22	11.6
	Carbohydrate (g)	8.75	13.7	−	−	8.38	12.7

* BSF = black soldier fly.

**Table 2:** Duration of black soldier fly larval development and survival to the prepupal stage when reared on different diets.

Study	Diet	Mean Duration (d = Day) of Larval Development ± SE	Percent Survival ^2^ ± SE
Myers et al. [14]	Cow (Dairy) Manure	30.4 ± 0.1	77.3 ± 4.55
Nguyen et al. [41] ^1^	Poultry Feed	23.0 ± 0.6	80.8 ± NA
	Pork Liver	22.5 ± 0.7	57.2 ± NA
	Swine Manure	34.0 ± 1.4	74.3 ± NA
	Kitchen Waste	23.8 ± 0.4	46.7 ± NA
	Fruits and Vegetables	28.7 ± 0.8	76.7 ± NA
	Fish Offal	26.5 ± 0.9	47.2 ± NA
Oonincx et al. [12] ^6^	HPHF	21 ± 1.4	86 ± 18.0
	HPLF	33 ± 5.4	77 ± 19.8
	LPHF	37 ± 10.6	72 ± 12.9
	LPLF	37 ± 5.8	74 ± 23.5
	Control	21 ± 1.1	75 ± 31.0
Current Study ^3^	Control (T1) ^4^	23.5 ± 0.4	87.0 ± 3.30
	(T2)	21.8 ± 0.4	
	7:35–55	45.3 ± 0.7	48.9 ± 3.30
	7:35–70	35.5 ± 0.7	57.8 ± 3.30
	21:21–55	38.4 ± 0.7	56.7 ± 3.30
	21:21–70 (T1) ^5^	32.3 ± 0.7	68.2 ± 1.96
	(T2)		56.0 ± 1.96
	35:7–55	45.6 ± 0.7	46.0 ± 3.30
	35:7–70	32.9 ± 0.7	52.9 ± 3.53

^1^ Larval development is the median amount of time taken to reach the prepupal stage. ^2^ SE is not presented for the study by Nguyen et al. [41], as the data are published as percent mortality. Data were transformed to percent survival for consistency. Additionally, the SE presented for the current study is the pooled SE calculated by JMP during the statistical analyses. ^3^ Treatments are presented as protein:carbohydrate-moisture. ^4^ Mean duration of larval development was significantly faster in Trial 2, but percent survival was not significantly different between trials. ^5^ Percent survival was significantly higher in Trial 1, but mean duration of larval development was not significantly different between trials. ^6^ HPHF: high protein, high fat, HPLF: high protein, low fat, LPHF: low protein, high fat, LPLF: low protein, low fat.

**Table 3:** Percent difference in black soldier fly larval development from three previous studies to data generated in the current study.

Study ^1^	Diet	Duration ^2^ (Days) of Larval Development ± SE	Percent Difference between Published Studies and Current Study ^3^ Data						
			Control	7:35–55	7:35–70	21:21–55	21:21–70	35:7–55	35:7–70
Myers et al. [14]	Cow (Dairy) Manure	30.4 ± 0.1	**−29%**	+39%	+15%	+23%	+6%	+40%	+8%
Nguyen et al. [41]	Poultry Feed	23.0 ± 0.6	**−2%**	+65%	+43%	+50%	+34%	+66%	+35%
	Pork Liver	22.5 ± 0.7	+0.6%	+67%	+45%	+52%	+36%	+68%	+37%
	Swine Manure	34.0 ± 1.4	**−40%**	+28%	+4%	+12%	**−5%**	+29%	**−3%**
	Kitchen Waste	23.8 ± 0.4	**−5%**	+62%	+39%	+47%	+30%	+63%	+32%
	Fruits and Vegetables	28.7 ± 0.8	**−24%**	+45%	+21%	+29%	+12%	+46%	+14%
	Fish Offal	26.5 ± 0.9	**−16%**	+52%	+29%	+37%	+20%	+53%	+21%
Oonincx et al. [12] ^4^	HPHF	21 ± 1.4	+7%	+73%	+51%	+59%	+43%	+74%	+44%
	HPLF	33 ± 5.4	**−37%**	+31%	+7%	+15%	**−2%**	+32%	**−0.4%**
	LPHF	37 ± 10.6	**−48%**	+20%	**−4%**	+4%	**−13%**	+21%	**−12%**
	LPLF	37 ± 5.8	**−48%**	+20%	**−4%**	+4%	**−13%**	+21%	**−12%**
	Control	21 ± 1.1	+7%	+73%	+51%	+59%	+43%	+74%	+44%
Mean difference			**−19%**	+48%	+25%	+32%	+16%	+49%	+17%

^1^ Each study used slightly different methods: Nguyen et al [41] conducted the experiment at 28 °C, Myers et al. [14] at 27 °C, and Oonincx et al. [12] at 28 °C. ^2^ Myers et al. [14] present data as mean time to complete larval development (d), Nguyen et al. [41] present data as median time (d) to reach the prepupal stage, and Oonincx et al. [12] present data as mean time (d) to collection of first prepupae. ^3^ Treatments are presented as Protein:Carbohydrate-Moisture. ^4^ HPHF: high protein, high fat, HPLF: high protein, low fat, LPHF: low protein, high fat, LPLF: low protein, low fat. Bold numbers indicate faster development in the current study.

**Table 4:** Percent difference in black soldier fly larval survival from three previous studies to data generated in the current study.

Study ^1^	Diet	Percent Survival ± SE	Percent Difference between Published Studies and Current Study ^2^ Data						
			Control	7:35–55	7:35–70	21:21–55	21:21–70	35:7–55	35:7–70
Myers et al. [14]	Cow (Dairy) Manure	77.3 ± 4.55	**+12%**	−45%	−29%	−31%	−22%	−51%	−37%
Nguyen et al. [41]	Poultry Feed	80.8 ± NA	**+7%**	−49%	−33%	−35%	−26%	−55%	−42%
	Pork Liver	57.2 ± NA	**+41%**	−16%	**+1%**	−1%	**+8%**	−22%	−8%
	Swine Manure	74.3 ± NA	**+16%**	−41%	−25%	−27%	−18%	−47%	−34%
	Kitchen Waste	46.7 ± NA	**+60%**	**+5%**	**+21%**	**+19%**	**+28%**	−2%	**+12%**
	Fruits and Vegetables	76.7 ± NA	**+12%**	−44%	−28%	−30%	−21%	−50%	−37%
	Fish Offal	47.2 ± NA	**+60%**	**+4%**	**+20%**	**+18%**	**+27%**	−3%	**+11%**
Oonincx et al. [12] ^3^	HPHF	86 ± 18.0	**+1%**	−55%	−39%	−41%	−32%	−61%	−48%
	HPLF	77 ± 19.8	**+12%**	−45%	−28%	−30%	−21%	−51%	−37%
	LPHF	72 ± 12.9	**+19%**	−38%	−22%	−24%	−15%	−44%	−31%
	LPLF	74 ± 23.5	**+16%**	−41%	−25%	−26%	−17%	−47%	−33%
	Control	75 ± 31.0	**+15%**	−42%	−25%	−28%	−19%	−48%	−35%
Mean difference			**+17%**	−16%	−6%	−7%	−2%	−19%	−11%

^1^ Each study used slightly different methods: Myers et al. [14] conducted the experiment at 27 °C, Nguyen et al. [41] at 28 °C, and Oonincx et al. [12] at 28 °C. ^2^ Treatments are presented as Protein:Carbohydrate-Moisture. ^3^ HPHF: high protein, high fat, HPLF: high protein, low fat, LPHF: low protein, high fat, LPLF: low protein, low fat. Bold numbers indicate greater survival in the current study.
